# Electrocardiographic changes in a rare case of flecainide poisoning: a case report

**DOI:** 10.1186/1757-1626-2-9137

**Published:** 2009-12-03

**Authors:** Andrea Rognoni, Marzia Bertolazzi, Marzia Peron, Sergio Macciò, Gemma Ternavasio Cameroni, Angelo Gratarola, Giorgio Rognoni

**Affiliations:** 1 Department of Cardiology, Sant'Andrea Hospital, Corso Mario Abbiate 21, 13100, Vercelli, Italy; 2 Emergency Department and Intensive Care Unit, Sant'Andrea Hospital, Corso Mario Abbiatr 21, 13100 Vercelli, Italy

## Abstract

Flecainide is a class Ic anti - arrhythmic drug with sodium channel blocking activities. We report a case of a 57 year - old woman who attempted a suicide by ingesting approximately 1,8 gr of flecainide. On the surface electrocardiogram this results in a large QRS complex and in prolongation of the QTc interval. Overdose with a class Ic drug is very uncommon, its management is difficult and the mortality high.

Because of a hemodynamic instability and in addition to supportive care and antidysrhythmics, she was treated with a high dose of sodium bicarbonate in hypertonic solution; after this infusion the patient's QRS progressive narrowed.

In conclusion, sodium bicarbonate may be useful in the treatment of widened QRS and to stabilize a overdose of class Ic anti - arrhythmic drugs.

## Introduction

Flecainide is a Vaughn - Williams class IC antiarrhtythmic agent used for the treatment of supra - ventricular and, also, ventricular arrhythmias.

In some countries, such as United states, its use is limited because of known proarrhytmic effects [1]. Chemically, it causes a rate - dependent slowing of a rapid sodium channels slowing phase of depolarisation [2]. Flecainide, also, slows conduction in all cadiac fibres, increasing conduction times in the atria, ventricles, atrio - ventricular node and his - Purkinje system and can cause myocardial depression. Flecainide is cleared mainly by the liver at a relatively high rate (5,6 ml/kg per minute) but its large value of distribution (4,9 ml(kg)) yields a large half - life of 11 hours [3]. Oral loading - dose 50 of flecainide is 50 - 498 mg/kg in rat; it is extensively metabolized mainly to m - O - dealkylated flecainide and the m - O - dealkylated lactam of flecainide; the first makes up to 20% of the drug's anti - arrhythmic activity. Furthermore flecainide is excreted mainly in urine (about 10 to 50% as the unchanged drug and the remainder as metabolites, depending on type of administration; about 5% is excreted in faeces).

Flecainide is a rare cause of suicide attempt by drug overdose; furthermore there are not specific antidote and no way of rapidly eliminating the drug from the body [4]. Commonly recommended therapies, including haemodialysis (in this case we can remove only 1% of unchanged flecainide), treatment with hypertonic saline solution and pacing, have not been shown to improve survival.

In the literature we find also some anecdotal case report of particularly therapy used to treat flecainide overdose. Timperly et al [5], in 2005, reported a case complicated by cardiogenic shock and treated with pharmacological inotropic support and intra - aortic balloon pump; in 48 hours both QRS and ventricular function had returned to normality.

Yasui et al [6], described, in a young woman, another possible approach such as peripheral cardiopulmonary bypass support (CBS) to maintain perfusion of the liver; this CBS successfully supported the patient until flecainide level decreased as a result of redistribution and normal clearance mechanisms.

We report a case of flecainide poisoning which was successfully treated with high dose of sodium bicarbonate in hypertonic solution.

## Case report

A 57 year - old women with a history of previous supra - ventricular tachyarrhythmia and chronic therapy with flecainide acetate (100 mg every day) without any cardiovascular risk factors, was admitted to our Emergency - Department having ingested 18 tablets of flecainide acetate (equivalent to approx 1,8 gr) for a suicide attempt.

On admission she was responsive and conscious; pulse was 90 bpm and blood pressure 110/70 mmHg.

**Figure 1:**
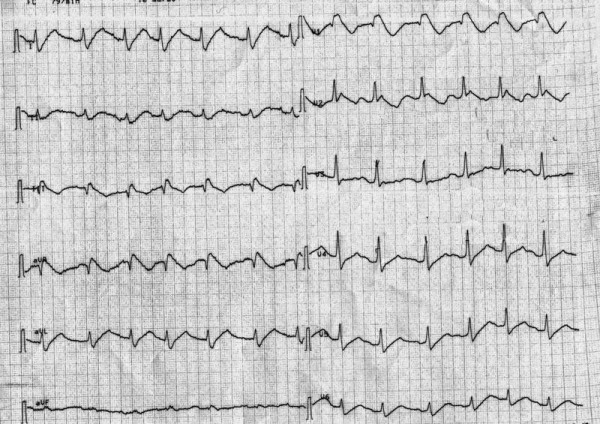
**The first electrocardiogram with a large QRS complex and a very long QTc interval**.

The first electrocardiogram (EKG) showed a sinusal rhythm with a large QRS complex (similar to a right blundle branch block) and a very long QT tract (about 500 msec.) (Figure 1).

The patient was carried into our Intensive Care Unit (ICU) and initially treated with oxygen and intravenous crystalloids. The results of the first arterial blood analysis were PH 7,472, PCO_2 _41,2 mmHg, PO_2 _130 mmHg (oxygen 4 l/minutes non invasive), HCO 29,8 ng/l. The hematochemical parameters were: K^+ ^3,5 mmol/l, Na^+ ^143 mmol/l, Cl^- ^103 mmol/l. Flecainide serum levels after 30 minutes from admission were 1940 μg/ml (therapeutic range are 200 - 1000 μg/ml).

Furthermore a therapy with instillation of 40 mg of activated charcoal and 40 mg of MgSO^4 ^was started. Approximately 45 minutes after admission to our ICU the poison Centre of "San Matteo Hospital" at Pavia (far off 60 Km) was contacted; they suggested bolus of sodium bicarbonate (NaCO_3_) 150 mEq and NaCO_3 _130 mEq in slow infusion (1 - 2 MEq/h) for 24 Hours.

Flecainide serum levels 6 hours after the beginning of this infusion were 990 μg/ml

**Figure 2:**
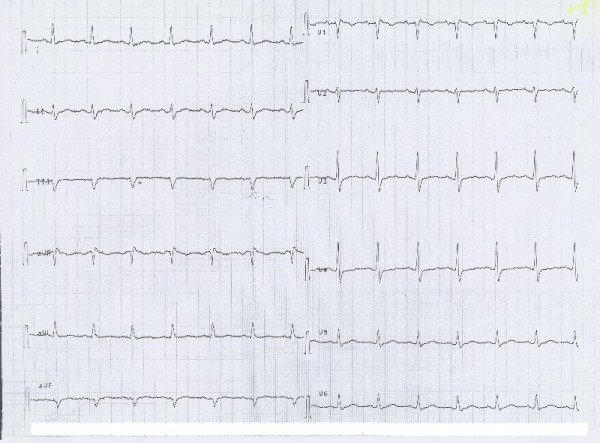
**The pre - discharge electrocardiogram**.

**Figure 3:**
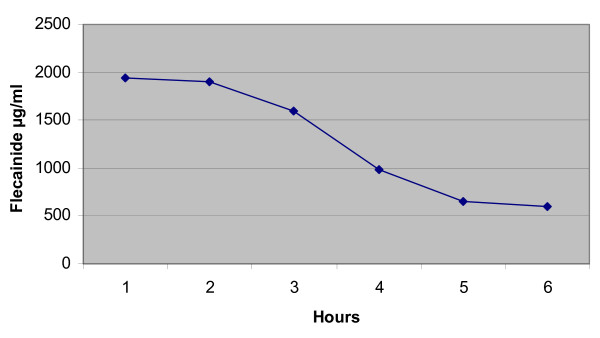
**Flecainide levels during hospitalization**.

Good progress were made over the next 72 hours and no signs of organ dysfunction were evident. None were apparent and the EKG had returned to normality and a psychiatric referral was made (Figure 2). The serum concentration of flecainide progressively returned to normal (Figure 3) and four days after the overdose the patient was transferred to medical ward and after other three days she was discharged to home.

## Discussion

As far as we are aware, there are no previously reported cases of high dose of sodium bicarbonate being used to treat acute flecainide overdose. This drug is a class Ic anthyarrhythmic agent which acts by blocking sodium channels involved in cardiac depolarization; this results in marked suppression of the cardiac conduction system in addition to a moderate negative inotropic effect [7]. All these actions are manifest on the EKC by increased lenght of PR, QRS and QTc intervals.

The pharmacokinetics qualities of flecainide are high oral bioavaibility of 95% elimination, half - life seems variable with a quarter of the drug undergoing renal excretion unchanged [8] the remainder undergoes hepatic metabolism to inactive derivates.

The therapeutic range of flecainide is considered to be 200 - 1000 μg/ml. In a toxic flecainide overdose, adverse cardiac effects including bradycardia, atrio - ventricular block, pulse less electrical activity and asystole can occur within 30 to 120 minutes of ingestion [9]. Treatment should be direct at decreasing gastrointestinal absorption through gastric emptying and administration of activate charcoal[9]; furthermore serum flecainide levels should be reduced through maintenance of organs perfusion which allows drug clearance and distribution.

In the literature many proarrhythmic effects of flecainide are described; they may be related to its promoting re - entry in ventricular issue [9].

Worsening of existing ventricular arrhythmias or the onset of ones can occur in up to 30% of patients [10]

Because type Ic drugs such as flecainide produce both therapeutic and toxic effects due to extensive rate - dependent sodium channel - blockade in the myocardium, attempt to treat toxicity with hypertonic sodium bicarbonate or molar lactate have been attempted in animals [11] and in humans [8,9,12] with mixed success.

Keyler et al [11] showed, in rats treated with 6 mEq/kg of hypertonic sodium bicarbonate, reduced to 26% flecainide induced QRS prolongation. Like - wise, 5 mEq/kg of hypertonic sodium bicarbonate in dogs resulted in significant shortening of the QRS and his - ventricular intervals within 10 minutes [13]. Furthermore the same authors reported that of in dogs with pacing - induced dysarrhythmias after flecainide administration, 6 of 7 responded to hypertonic sodium bicarbonate, with only 1 of 7 responding to placebo[13]. Hypertonic sodium bicarbonate and sodium clorate work by increasing the extra cellular concentration of sodium displacing flecainide from its receptors sites either inside the selectivity filter of the fast sodium channels or at on external anaesthetic receptors site [14].

Because flecainide is a weak acid with a high pKa, alkalinization may also decrease the active - ionized fraction of flecainide necessary for sodium channels blockade. Chouty et al [15] used doses of 500 mL of 1 M sodium lactate endovenous for more than 30 minutes to treat three cases of flecainide poisoning with rapid narrowing of the QRS interval and correction of hypotension, and another flecainide overdose responded to combination to a combination of 250 mmol of hypertonic sodium bicarbonate, 136 mmol NaCl and physostigmine 2 mg endovenous in the report of Wilkelman [9].

Our patient's dysarrhythmia did not resolve until sodium bicarbonate was administered.

All our observations suggest that sodium bicarbonate may be useful for the treatment of widened QRS and ventricular ectopy resulting from flecainide toxicity.

## Consent

Written informed consent was obtained from the patient for publication of this case report and accompanying images. A copy of the written consent is available for review by the Editor-in-Chief of this journal.

## Competing interests

The authors declare that they have no competing interests.

## Authors' contributions

AR carried out the research about the effects of flecainide; MB partecipated in the study of the sides effects of flecainide; MP performed the layout of the figures; GTC partecipated in the writing of the text; AG partecipated in the writing of the text; GR partecipated in the writing of the text. All authors read and approved the final manuscript.
